# Comparative analysis of alkaline-extracted hemicelluloses from Beech, African rose and Agba woods using FTIR and HPLC

**DOI:** 10.1016/j.heliyon.2022.e09714

**Published:** 2022-06-11

**Authors:** Victoria Ezinne Ottah, Arinze Linus Ezugwu, Tobechukwu Christian Ezike, Ferdinand Chiemeka Chilaka

**Affiliations:** Department of Biochemistry, University of Nigeria, Nsukka Enugu State, Nigeria

**Keywords:** Wood, Hemicellulose, Heteropolysaccharide, FT-IR, HPLC

## Abstract

The vast application of hemicellulose in industry is greatly influenced by its chemical components. The current study focuses on identifying the chemical components of a high yield alkaline-extracted hemicellulose and characterization to serve as a guide for more specific and effective biotechnological uses. In this study we isolated hemicellulose from sawdust of three different wood species (Beech, African rose and Agba woods) and characterized them using FTIR and HPLC techniques. Hydroxyl spectra vibrations were observed at 3919-3671 cm^−1^ and 3454-3211 cm^−1^ which indicates the presence of non-hydrogen bonded OH stretch and normal polymeric OH stretch in all three samples. The samples contained residual lignin indicated by IR absorption bands at 1592 and 1525 cm^−1^. The presence of C=O stretching vibrations of acetyl groups at 1734 cm^−1^ indicated that African rosewood was generally an acetylated molecule. Each heteropolysaccharide also contained reducing monosaccharides at their ends suggested by the C–H stretching vibrations. Infrared absorptions characteristic of asymmetric β-1,6-glycosidic stretching was present in Beechwood and Agbawood, respectively, and African rosewood gave three absorption bands β-1,3-glycosidic stretch, β-1,4-glycosidic stretch and an asymmetric β 1,6-glycosidic stretch, respectively. Agbawood gave a major absorption band at 923.75 cm^−1^ corresponding to the absorption band at β-1,4-glycosidic stretching. African rosewood contained 96 % mannose and 4 % of an unidentified sugar. Beechwood contained primarily glucose, but Agbawood contained 20, 14, 8 and 57 % glucose, mannose, galactose, and an unidentified sugar, respectively.

## 1 Introduction

Hemicellulose is a major structural polysaccharide of the plant cell wall along with cellulose and lignin (Collins et al., 2005; Sheller and Ulvskov, 2010; Wang et al., 2020). They are ideal sources of energy although greatly underutilized. Mostly, hemicellulose is a heteropolysaccharide composed of β-1,4-linked-D-xylose monomers connected with side branches of residues of arabinosyl, glucuronosyl, acetyl, uronyl, and mannosyl (Singh et al., 2003; Protyusha and Amit, 2018). In hardwoods, they occur mainly as O-acetyl-4-O-methylglucuronoxylan and in softwood they occur as arabino-4-O-methylglucuronoxylan (Wang et al., 2020). Recently, tailored research on the use of hemicelluloses in the production of composites with more desirable properties are ongoing (Chang et al., 2018).

One major challenge with recovery and utilization of the components of lignocellulose is its recalcitrance. Hence, pre-treatment is very important to separate the individual components prior to any biotechnological application (Merino and Cherry, 2007; Brodeur et al., 2011). The idea of pre-treatment is to produce highly digestible polysaccharides, avoid degradation of sugars, minimize the formation of inhibitors, recover lignin for conversion into valuable coproducts, and be cost effective in relation to chemical and energy requirements (Merino and Cherry, 2007; Demirbas, 2009). Methods used to achieve this include physical, biological, chemical, and physicochemical. However, physical pre-treatments have very high energy requirement depending on the final particle size and often the required energy is higher than the theoretical energy content available in the biomass (Brodeur et al., 2011). Biological pre-treatments involve the use of microorganisms. However, even though they are milder and of low cost, they take longer time, and the organism may end up degrading the entire biomass (Shi et al., 2008). Chemical pre-treatments involve the use of alkali and/or acid to disrupt the lignin structure and allow recovery of its components. Physicochemical pre-treatments refer to steam explosion techniques that combines both chemical and physical methods to break the structure of the lignocellulosic material mostly using very high temperatures ranging from 190 °C to 270 °C, which of course would degrade some of the water-soluble sugars (Viola et al., 2008; Wang et al., 2010). Acid pre-treatment is generally used for digestion but not for fractionation of the individual components of lignocellulose (Marzialetti et al., 2008; Zhang, 2008) because they could result in the hydrolysis of some of the lignocellulose sugars and result in a significant reduction in its yield. However, the conditions for alkaline pre-treatment are usually less severe than physicochemical and acid pre-treatments as they require lower temperatures and reduce the amount of sugar loss by hydrolysis (Junior et al., 2011).

Beechwood, African rosewood and Agba wood are commonly used materials in building and construction. These woods generate a large amount of wastes every year as sawdust. These by-products are potentially valuable materials that can be used for various biotechnological processes such as biofuel, enzyme, xylooligosaccharides production and in the construction of biomaterials (Chakdar et al., 2016; Chang et al., 2018; Chinga-Carrasco, 2018).

Fourier transform infrared spectroscopy (FTIR) is an important tool in analytical chemistry with vast applications especially for polymers and organic compounds which reveals the composition of materials whether in the solid, liquid of gaseous state (Fanesi et al., 2019; Lv et al., 2015; Rana et al., 2010; Hori and Sugiyama, 2003; Biagini and Tognotti, 2003). It uses Fourier transform process to convert data from a sample interferogram into an infrared spectrum of absorption or transmission (Introduction to FT-IR sample handling, 2013). By this process, both organic and inorganic compounds present within the sample are identified by the recognition of specific molecular groups according to the IR absorption frequency (Mohamed and Mohamed, 2019; Lv et al., 2015; Bian et al., 2010; Morales-Ortega et al., 2013; Hori and Sugiyama, 2003).

High performance liquid chromatography is a popular analytical technique used to separate, identify, and quantify the components of a mixture (Mukthi, 2016; Crowley, 2020). This technique has been used extensively for the identification and quantification of the monomers present in hydrolysates of cellulose, hemicelluloses, and lignin based on their molecular weight distribution (Morales-Ortega et al., 2013; Wang et al., 2020; Crowley, 2020). Whether the polysaccharide is to be used for enzyme production, production of biofuel or biomaterials, the chemical composition of the material greatly influences its solubility, physical and mechanical properties (Chakdar et al., 2016).

In the present study, the Fourier transform infrared spectroscopy tool was used for the identification and verification of hemicellulose extracted from Beech, African rose and Agba woods by alkaline extraction method. HPLC was used to analyse hydrolysates for identification and quantification of the constituting monosaccharides.

## 2 Methods

### **2.1** Alkaline extraction of hemicellulose from lignocellulosic samples

This was done by modifying the method described by Peng et al. (2011). Milled lignocellulosic biomass powders (saw dust) obtained from Timber-processing site, Nsukka, were soaked in 10 % NaOH with a ratio of 1:10, and kept overnight in an incubator shaker with agitation (120 rpm) at 60 °C. The mixture was boiled at 100 °C for 3 h. After alkaline treatment, the supernatant was recovered by filtration. Solubilized hemicellulose was precipitated in 1:2 volume of analytical grade ethanol (99.8 %). The precipitate was recovered by decantation, oven-dried at 60 °C, weighed, crushed into powder, and stored in an air-tight container at room temperature. The percentage yield of the extracted hemicellulose was calculated as follows (equation 1):

(1) $$Percentage\;yield\;\left(\%\right)=\frac{dry\;weight\;of\;extracted\;hemicellulose\;\left(g\right)}{dry\;weight\;of\;sample\;\left(g\right)}\times 100$$

### 2.2 Fourier-transform infrared (FTIR) spectroscopic analysis

The extracts from Beech, African rose and Agba wood were analysed as dry samples in an FTIR apparatus over a spectra wavelength of 4000 to 500 cm^−1^ at a resolution of 4 cm^−1^ and an interval of 1 cm^−1^ at room temperature according to the method of Lal et al. (2010).

### 2.3 Hydrolysis of hemicellulose

Hemicellulose hydrolysis was carried out under acidic conditions as described by Fanesi et al. (2019). Dried samples (0.5 g) were dissolved in 1 ml of water. To aliquots of sample (500 μL), 1ml of H_2_SO_4_ (98 % v/v) was added and hydrolysis was carried out at 80 °C for 1 h with mixing. After acid hydrolysis, 100 μL aliquots was taken from the mixture and put into clean tubes and diluted to 1 ml with distilled water. Calcium carbonate was used to neutralize the pH to 5–6 and the mixture was filtered before analysis.

### 2.4 Monosaccharide identification and quantification of hydrolysates by HPLC

Aliquots (10 μL) of each neutralized hydrolysate sample were analysed by HPLC (Shimadzu) with quantification referenced to xylose, glucose, galactose, mannose, and arabinose standards. Separation was carried out with distilled water as mobile phase at a flow rate of 0.4 ml/min using refractive index detector at 50 °C.

## 3 Results

### 3.1 Alkaline extraction of hemicellulose from lignocellulosic samples

Saw dust from different woods collected from timber – processing sites gave yields of 45.5, 39.7 and 36.7 % for African rosewood, Beechwood and Agbawood sawdust, respectively (Table 1).

**Table 1:** Alkaline extraction of hemicellulose from sawdust of Beech, African rose and Agba woods.

Lignocellulosic sample	Weight of Sample (g)	Weight of Extract (g)	Hemicellulose Yield (%)
Beechwood	240	95.3	39.7
African rosewood	120	54.6	45.5
Agbawood	120	44.4	36.7

### 3.2 FTIR analysis of hemicellulose

**Figure 1:**
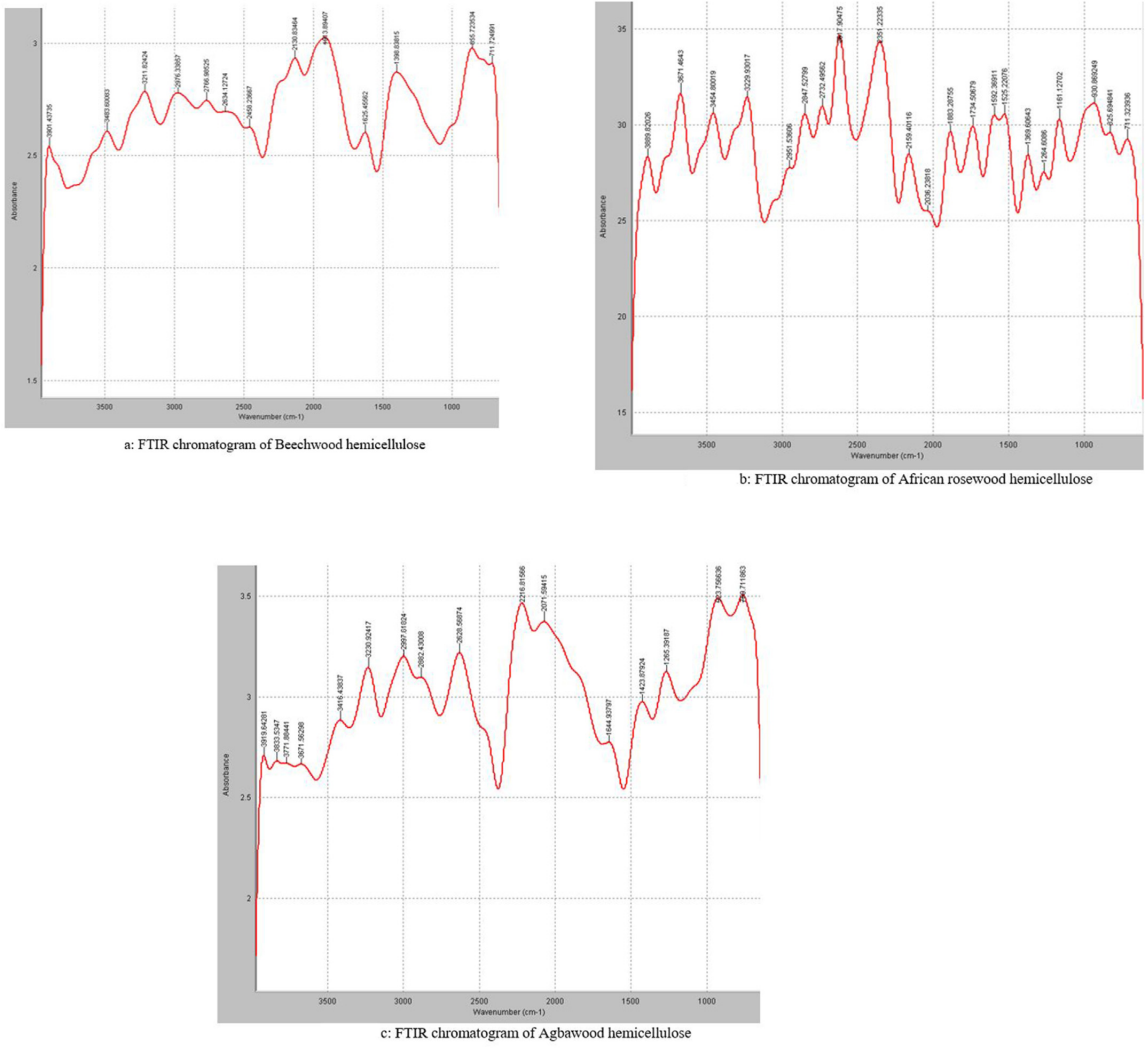
FTIR chromatogram of a) Beechwood, b) African rosewood and c) Agbawood hemicellulose.

Absorption at the infra-red regions of 4000 to 1500 cm^−1^ indicating non-hydrogen bonded O–H stretching, normal polymeric OH-stretching, C–H_2_ stretching vibrations, C–H stretching, C–O stretching, C=O stretching, and C–H deformation was observed for extract from Beech (Figure 1a), African rose (Figure 1b), and Agba woods (Figure 1c). In the fingerprinting region for infrared absorption bands characteristic of saccharides (1200–800 cm^−1^), one singular peak was observed at 855.72cm^−1^ for Beech wood (Table 2) indicating the presence of an asymmetric β-1,6-glycosidic stretching, African rosewood showed three absorption bands at 1161.1, 930.8 and 825.6 cm^−1^ (Table 3) indicating an asymmetric β-1,3-glycosidic stretch, β-1,4-glycosidic stretch and an asymmetric β-1,6-glycosidic stretch, respectively. Agbawood gave a major absorption band at 923.75 cm^−1^ (Table 4) corresponding to the absorption band of β-1,4-glycosidic stretching.

**Table 2:** FTIR analysis of Beechwood hemicellulose.

Wave number (cm^−1^)	Functional group
3901.43	Non-hydrogen bonded O–H stretching
3483.60, 3211.82	Normal polymeric O–H stretching
2976.33	C–H_2_ stretching symmetric vibrations
2766.98, 2634.12	C–H stretching (aldehydes)
2458.23	C–O stretching
2130.83, 1913.81	C–H bending
1625.45	C–H deformation
1398.83	O–H deformation
**Fingerprint region**	
**Wave number (cm**^**−1**^**)**	**Bond present**
855.72	Asymmetric β-1,6-glycosidic stretching
711.725	O–H bending out of plane

**Table 3:** FTIR analysis of African rosewood hemicellulose.

Wave number (cm^−1^)	Functional group
3889.8, 3671.4	Non-hydrogen bonded O–H stretching
3454.6, 3229.9	Normal polymeric O–H stretch
2951.5, 2847.5	C–CH_2_ stretching
2617.9	C–H stretching
2351.2	C–O stretching
2159.4, 2036.2, 1883.2	C–H bending
1734.5	C=O (Ketones and ester carbonyl groups)
1592.3, 1525.2	C–C=C stretching of aromatic rings
1264.6, 1369.6	C–O stretch
**Fingerprint region**	
**Wave number (cm**^**−1**^**)**	**Bond present**
1161.1	Asymmetric β-1,3-glycosidic stretching
930.8	β-1,4-glycosidic C–O–C stretching
825.6	Asymmetric β-1,6-glycosidic stretching
711.3	O–H bending out of plane

**Table 4:** FTIR analysis of Agbawood hemicellulose.

Wave number (cm^−1^)	Functional group
3919.64, 3833.53, 3771.88, 3671.56	Non-hydrogen bonded O–H stretching
3416.46, 3230.92	Normal polymeric O–H stretching
2997.61, 2882.43,	C–CH_2_ stretching
2628.56	C–H stretching
2216.81, 2071.59	C–H bending
1644.93	C=O stretching
1423.87	CH_2_ bending
**Fingerprint region**	
**Wave number (cm**^**−1**^**)**	**Bond present**
1265.39	Asymmetric β-1,3-glycosidic stretching
923.75	β-1,4-glycosidic C–O–C stretching
759.71	O–H bending out of plane

### 3.3 HPLC monosaccharide characterization of samples

**Figure 2:**
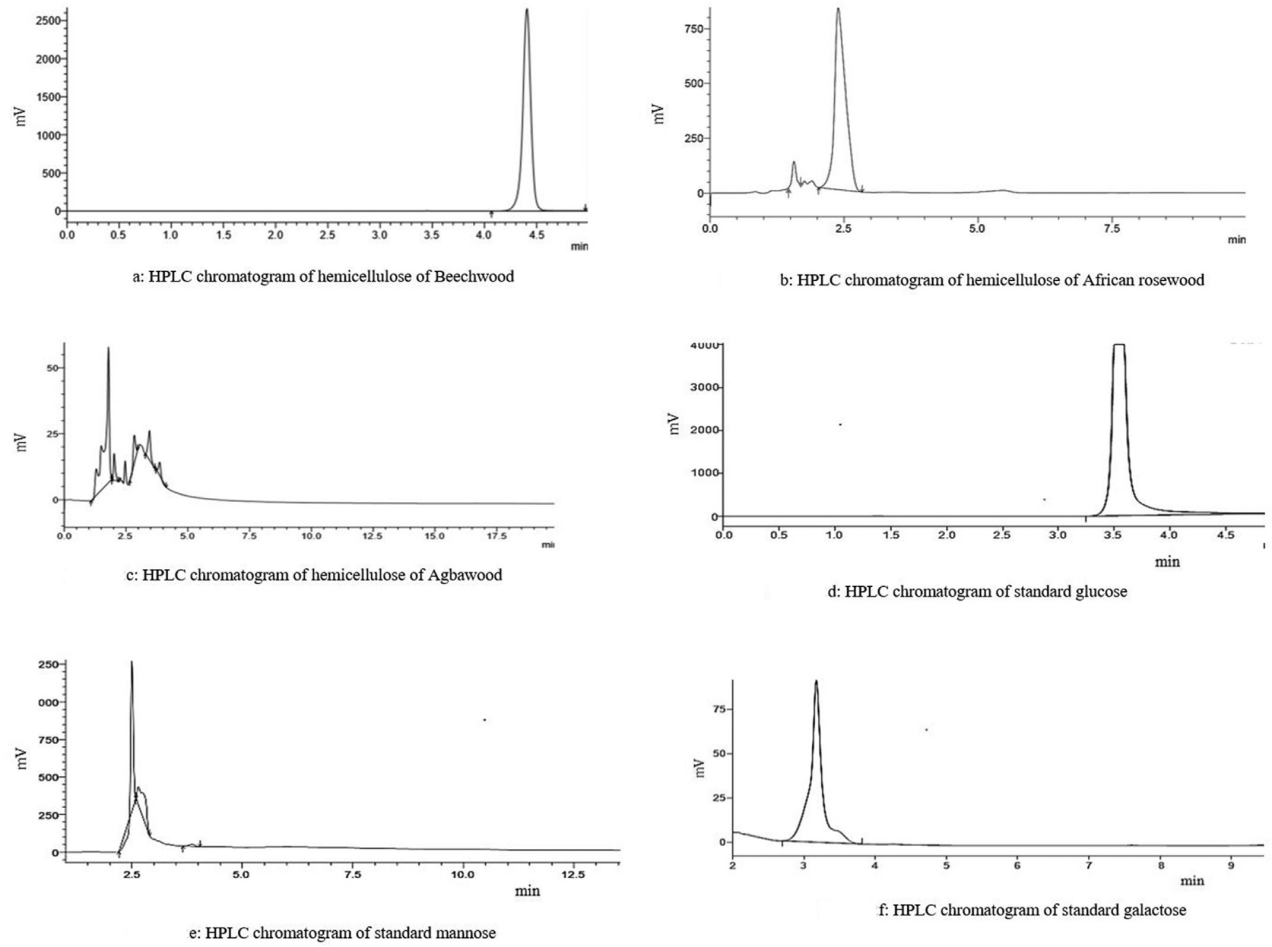
HPLC chromatogram of hemicellulose of a) Beechwood, b) African rosewood, c) Agbawood, d) Standard glucose e), Standard mannose, f) Standard galactose.

The monosaccharide compositions and their individual concentrations shown in Tables 5 and 6 indicated that Beech wood was composed majorly of glucose (RT = 4.402 min) (Figure 2a & 2d). African rose wood comprised majorly mannose (RT = 2.383min) (Figure 2b & 2e), whereas Agba wood comprised 14 % mannose (RT = 1.7 and 2.0 min), 9.22 % galactose (RT = 2.8 min) (Figure 2c, 2e & 2f).

**Table 5:** Monosaccharide composition of extracted hemicelluloses from Beech, African rose and Agba woods.

Sample	Retention time (min)	Range	Identified sugar
**Beechwood xylan**	4.402	4.08–4.98	Glucose
**African rosewood xylan**			
Peak 1	1.65	1.5–1.7	Unidentified
Peak 2	2.38	2.0–2.8	Mannose
**Agbawood xylan**			
Peak 1	1.7	1.1–1.9	Unidentified
Peak 2	2.0	1.9–2.4	Mannose
Peak 3	2.6	2.5–2.7	Mannose
Peak 4	2.8	2.7–3.0	Galactose
Peak 5	3.4	3.3–3.7	Glucose
Peak 6	3.85	3.7–4.7	Glucose
**Standards**			
Mannose	2.49	2.21–2.7	
Galactose	3.16	2.7–3.8	
Glucose	3.54	3.25–4.98	
Arabinose	5.99	5.25–7.25	
Xylose	7.33	6.8–7.9

**Table 6:** HPLC monosaccharide peak table of extracted hemicelluloses from Beech, African rose and Agba woods.

Samples	Area (mV/min)	% Monosaccharide concentration
**Beechwood xylan**	1144	100
**African rosewood xylan**		
Peak 1	´14	4
Peak 2	336	96
**Agbawood xylan**		
Peak 1	22.8	56.47
Peak 2	4.5	11.14
Peak 3	1.35	3.34
Peak 4	3.6	8.91
Peak 5	5.0	12.38
Peak 6	3.12	7.72

## 4 Interpretation

Hemicellulose has been reported to be found in large quantities in hardwoods (15–30) (Collins et al., 2005). Junior et al. (2011) reported 42 % yield from Eucalyptus woods under extreme alkaline conditions of 20 % NaOH treatment at 100 °C for 60 min, whereas treatment of wood wastes from African rosewood, Beechwood and Agbawood using 10 % NaOH at 60 °C overnight was sufficient to dislodge the lignin barriers, cause cellulose swelling and release the hemicellulose with a yield as high as 45.5, 39.7 and 36.7 % respectively. Alkaline extraction under mild conditions have so far proven to be the more efficient in extracting hemicelluloses from lignocellulose as it prevents the hydrolysis of the water-soluble fractions that happens at very high temperatures (up to 270 °C) and high pressures (Viola et al., 2008; Wang et al., 2010). Acid hydrolysis could occur with the use of acid for extraction under extreme conditions, thus reducing the yield. Zhao et al. (2008) showed the effectiveness of sodium hydroxide pre-treatment for hardwoods, wheat straw, switchgrass, and softwoods removing more than 74 % of the lignin content.

The hydroxyl spectra vibrations observed in the three samples confirmed the presence of the heteropolysaccharides. Hemicelluloses are largely soluble in water; their solubility can be attributed to the presence of hydroxyl groups as these OH groups interact with water molecules forming hydrogen bonds (Sun et al., 2004). The IR spectra at 1592 and 1525 cm^−1^ is characteristic of aromatic rings like benzene, suggesting that there is residual lignin on the extracted hemicellulose (Chan et al., 2013). The presence of a band at 1734 cm^−1^ represents C=O stretching. The C=O stretching band could have occurred because of one of the following: first it could indicate the presence of an acetyl groups on the heteropolysaccharide, representing an acetylated molecule, or it could suggest that a ketone sugar such as fructose could be one of the substitutions within the molecule (Bian et al., 2010). The presence of C–H stretch of aldehydes suggests the presence of reducing monosaccharides at the end of the polysaccharide chain. Generally, bands absent around 1720 cm^−1^ tells us that the ketone carbonyls, glycosidic bonds, and hydroxyl groups were not oxidized. Thus, we can say that the attack on the glycosidic linkages during the alkaline method of extraction was minimal (Magaton et al., 2008).

The fingerprinting region indicate infra-red absorption patterns for specific bond types in the polysaccharide, ranging between 1200 cm^−1^ to 800 cm^−1^ (Egüés et al., 2014; Morales-Ortega et al., 2013), which concerns mainly glucans and mannans (825, 855, 930, 923, 1161 cm^−1^). For Beechwood, the presence of an asymmetric β-1, 6-glycosidic stretch was confirmed. African rose wood showed three absorption bands at 1161.1 cm^−1^, 930.8 cm^−1^, 923.7 cm^−1^ indicating an asymmetric β-1, 3-glycosidic stretch, β-1, 4-glycosidic C–O–C stretch and an asymmetric β-1,6-glycosidic stretch, respectively. Agba wood gave a major absorption band at 923.75 cm^−1^ corresponding to the absorption band at β-1, 4-glycosidic C–O–C stretch (Barron and Rouau, 2008).

Although FTIR gives us information on the types of bonds present in the polysaccharide chain, it does not tell us the type of sugars that make up the polysaccharide. This is especially important because it gives us structural information on the type of heteropolysaccharide and the nature of the backbone. Altogether, a good understanding of both the individual monosaccharides present as well as how they are attached go a long way in enhancing the industrial application of the enzyme product. The elution profiles of these sugars are determined by their retention time (RT). Sugars with higher molecular weights elute first while those with lower molecular weights elute later. Beech wood showed 100 % similarity (RT = 4.402 min) with reference to glucose standard. African rose wood majorly mannose (RT = 2.383 min) and one unidentified sugar. Agbawood comprised 56.47 % of an unidentified sugar, 14 % mannose (RT = 1.7 and 2.0 min), 9.22 % galactose (RT = 2.8 min) and 20.82 % glucose units (RT = 3.4 and 3.8 min), respectively. The unidentified sugar could be one of the following: either a monosaccharide not included among the standard sugars used, or partial/incomplete hydrolysis of the polysaccharide gave rise to either a disaccharide or an oligosaccharide. Because of the rapid elution indicated by shorter retention time, it could be inferred that the unidentified sugars possess higher molecular weight than the other monosaccharides, which further corroborates that the sugar could be a disaccharide or oligosaccharide resulting from incomplete hydrolysis.

## 5 Conclusion

From this experiment, it was observed that milder alkaline treatment of lignocelluloses under less extreme conditions (60 °C, atmospheric pressure) was sufficient for hemicellulose extraction with very little hydrolysis of the water-soluble fractions. Hemicelluloses from Beech, African rose and Agba woods varied in chemical composition, thus, their industrial applications will vary depending on the peculiar utility in mind.

## Declarations

### Author contribution statement

Victoria Ezinne Ottah: Conceived and designed the experiments; Performed the experiments; Analyzed and interpreted the data; Contributed reagents, materials, analysis tools or data; Wrote the paper.

Arinze Linus Ezugwu, Tobechukwu Christian Ezike: Contributed reagents, materials, analysis tools or data.

Ferdinand Chiemeka Chilaka: Conceived and designed the experiments; Analyzed and interpreted the data; Contributed reagents, materials, analysis tools or data.

### Funding statement

This research did not receive any specific grant from funding agencies in the public, commercial, or not-for-profit sectors.

### Data availability statement

Data will be made available on request.

### Declaration of interests statement

The authors declare no conflict of interest.

### Additional information

No additional information is available for this paper.
